# A Panel of Plasma Exosomal miRNAs as Potential Biomarkers for Differential Diagnosis of Thyroid Nodules

**DOI:** 10.3389/fgene.2020.00449

**Published:** 2020-05-19

**Authors:** Meihua Liang, Siming Yu, Shuli Tang, Lu Bai, Jianan Cheng, Yuanlong Gu, Shuang Li, Xin Zheng, Lian Duan, Liang Wang, Yanqiao Zhang, Xiaoyi Huang

**Affiliations:** ^1^Department of Endocrinology, The Second Affiliated Hospital of Harbin Medical University, Harbin, China; ^2^Department of Pharmacy, Drug Clinical Trails Institution, Peking University Shenzhen Hospital, Shenzhen, China; ^3^Department of Gastrointestinal Medical Oncology, Harbin Medical University Cancer Hospital, Harbin, China; ^4^Biotherapy Center, Harbin Medical University Cancer Hospital, Harbin, China; ^5^Hematology Oncology, Taizhou Municipal Hospital, Taizhou, China; ^6^College of Bioinformatics Science and Technology, Harbin Medical University, Harbin, China; ^7^Moffitt Cancer Center, Tampa, FL, United States

**Keywords:** thyroid cancer, thyroid nodule, exosome, miRNA, liquid biopsy, cancer diagnosis

## Abstract

**Background:** A liquid biopsy using circulating exosomal genetic materials provides new insights for thyroid cancer diagnosis. This study aimed to identify plasma-derived exosomal biomarkers that could be used for early detection of papillary thyroid carcinoma (PTC).

**Method:** Exosomal miRNAs in plasma were isolated from patients with benign thyroid nodules and patients with PTC. Profiling of exosomal miRNA was performed using RNA sequencing (RNA-seq) to identify miRNA candidates and differentiate the benign from malignant. The validation cohort consisted of 30 patients with benign thyroid nodules, 35 PTC patients, and 31 healthy individuals. Real-time PCR was used to quantify the expression of miRNA candidates. The diagnostic potential of the candidates was evaluated by receiver operating characteristic (ROC) curves.

**Results:** After RNA-seq, eight plasma exosomal miRNAs were selected as candidates. Further validation indicated that the levels of exosomal miR-16-2-3p, miR-223-5p, miR-34c-5p, miR-182-5p, miR-223-3p, and miR-146b-5p were significantly lower in nodules compared to healthy controls (*p* < 0.0001), whereas miR-16-2-3p and miR-223-5p were significantly higher in the PTC cases than in those with benign nodules (*p* < 0.05). ROC analyses revealed that the above six miRNAs were potent indicators for detection of thyroid nodules. Meanwhile, miR-16-2-3p and miR-223-5p can be utilized for detecting PTC from benign nodules. Additionally, combined miRNA panels showed increased diagnostic sensitivities and specificities compared to single miRNA markers.

**Conclusion:** Six aberrantly expressed plasma exosomal miRNAs may be used as diagnostic biomarkers to differentiate thyroid nodules from healthy individuals. The panel consisting of miR-16-2-3p, miR-223-5p, miR-101-3p, and miR-34c-5p are eligible for discriminating benign from malignant thyroid nodules.

## Introduction

Thyroid nodules are one of the most frequently diagnosed pathologies in diseases of the endocrine system. Studies have shown that up to 70% of randomly selected individuals bear nodular disease, and is more common in women and among older people. Although 90% of thyroid nodules are benign, and are hyperplastic nodules of a multinodular goiter, ~7–15% of all ultrasound-detected nodules are diagnosed as thyroid cancer (TC) (Hegedus, 2004). The incidence of TC, especially papillary thyroid carcinoma (PTC), has increased dramatically in many countries, implying that the need to easily and accurately distinguish the benign nodules from PTC is urgent (Haugen et al., 2016). Ultrasound is the most prevalent option to screen thyroid nodules in physical examination, but accurate diagnosis requires a highly skilled operator. Likewise, fine needle aspiration (FNA) biopsies performed under ultrasound guidance is the most sensitive mainstay for characterizing thyroid nodules. However, FNA is an invasive diagnostic method and is heavily dependent on the technical performance and experience of the operators. Recently, liquid biopsy has emerged as a promising option to develop non-invasive and sensitive biomarkers for various malignancies, including PTC.

MicroRNAs (miRNAs) are small and non-coding RNAs that regulate the expression of multiple protein-coding genes at a post-transcriptional level by negatively regulating gene expression. Alteration of miRNA expression is often involved in the development and progression of different human diseases, implying that miRNAs hold diagnostic and prognostic potentials in clinic (Keshavarzi et al., 2017; Mirzaei, 2017; Gholamin et al., 2018; Mirzaei et al., 2018). To the end of fully utilizing miRNAs to develop non-invasive means of diagnosis, exosomal miRNAs in circulation are suitable for developing diagnostic biomarkers because of their enhanced stability and selective miRNA enrichment (Jin et al., 2017). To date, few studies have profiled plasma exosomal miRNAs and identified specific miRNA biomarkers for diagnosis of thyroid nodules as well as for characterization of PTC.

Circulating exosomes in the blood are small (30–100 nm) membrane particles that are released into the extracellular environment through fusion of multivesicular bodies with the plasma membrane. The outer lipid bilayer membrane of the extracellular vesicle protects its contents from being degraded while circulating throughout the body. Exosomes are reported to contain functional biomolecules, such as mRNA and miRNA that can be horizontally transferred to recipient cells and thereby carry out their preserved roles (Colombo et al., 2014). Importantly, the protected and selectively enriched biomolecules in exosomes also offer an opportunity to develop cancer-specific diagnostic, prognostic, or even therapeutic biomarkers. For example, miRNA-31 was found to be over-represented in the plasma exosomes of patients with PTC when compared to follicular TC (Samsonov et al., 2016). It is important to note that the majority of the studies focusing on circulating miRNA biomarkers for PTC used whole serum or plasma as a starting material, rather than the highly stable circulating exosomes. For example, serum let-7e, miR-151-5p, and miR-222 were significantly increased in PTC cases compared to benign cases and healthy controls (Yu et al., 2012). High levels of serum miR-146b, miR-222, miR-221, miR-124-3p, miR-9-3p, miR-25-3p, and miR-451a, and low levels of serum miR-196b-5p distinctively characterize PTC (Li et al., 2015; Yu et al., 2016; Zhang et al., 2017; Jahanbani et al., 2018). Likewise, miR-190 was upregulated, whereas miR-95, miR-579, and miR-29b were downregulated in PTC compared with healthy subjects and patients with nodular goiters (NG) (Cantara et al., 2014). Another group determined the expression levels of circulating miR-146b and miR-155 to discriminate between benign and malignant lesions (Lee et al., 2015). The potential of miR-222 and miR-146b levels in the circulation as tumor biomarkers has also been proven to improve patient stratification regarding the risk of recurrence (Lee et al., 2013). These results suggest that circulating miRNAs may aid in the differential diagnosis of thyroid nodules, but the clinical prospects of exosome-enclosed miRNAs from plasma for PTC and benign nodules has not been fully investigated.

Our group has previously developed a data-generation and data-analysis pipeline for exosomal miRNA profiling, including isolation of plasma exosome, RNA extraction, library preparation, sequencing, and bioinformatics analyses. To identify exosomal miRNAs and estimate their diagnostic potential for discriminating thyroid nodules, we applied this pipeline to examine plasma exosomal miRNA profiles in healthy subjects (HS), benign nodular goiters (NG), non-metastatic PTC (nmPTC), and metastatic PTC (mPTC). The purpose of this study was to identify plasma-derived exosomal miRNA biomarkers that could be used as a non-invasive method with high specificity and sensitivity for malignant thyroid nodule diagnosis at early stages.

## Materials and Methods

### Patients and Samples

The patients (discovery stage, *n* = 24; validation group, *n* = 65) were prospectively recruited at the Department of Thyroid Surgery of the Second Affiliated Hospital of Harbin University (Heilongjiang, China). Among them, 16 PTC patients (eight with mPTC and eight nmPTC) and eight benign NG individuals enrolled in our discovery stage. Another 35 PTC patients (19 with mPTC and 16 nmPTC) and 30 benign NG patients were involved in the validation stage. The inclusion criteria were as follows: all patients were newly diagnosed with PTC or NG, which was independently confirmed by two pathologists post-thyroidectomy. Patients with metabolic, immune, or blood-related diseases, or those subjected to any medical treatment before sample collection were excluded. Thyroid cancer was staged according to the International Union against Cancer (UICC) guidelines. The 31 control HS in validation sets were age- and gender-matched healthy individuals with no current or previous malignancy or thyroid disease. Detailed clinical information is summarized in Table 1. This study was carried out in accordance with the recommendations of the Clinical Research Ethics Committee (CREC) of the Harbin University Cancer Hospital. The protocol was approved by the CREC (KY2016-11). All subjects gave written informed consent in accordance with the Declaration of Helsinki.

**Table 1:** Clinical characteristics of the patients and healthy individuals of all sets.

**Categories**	**Discovery set (*****N*** **=** **24)**			**Validation set (*****N*** **=** **96)**			
	**NG**	**nmPTC**	**mPTC**	**NG**	**nmPTC**	**mPTC**	**HS**
	**8**	**8**	**8**	**30**	**16**	**19**	**31**
**Gender**							
Male/Female	2/6	2/6	2/6	15/15	8/8	10/9	17/14
Age (Average ± SD)	44.1 ± 5.6	43.6 ± 6.7	44.4 ± 4.0	46.3 ± 10.9	45.4 ± 6.3	43.2 ± 5.9	45.7 ± 11.0
**TNM stage**							
T0/T1	8/0	0/8	0/8	30/0	0/16	0/19	NA
N0/N1	8/0	8/0	0/8	30/0	16/0	0/19	NA
M0/M1	8/0	8/0	8/0	30/0	16/0	19/0	NA

*NG, Benign nodular goiters; nmPTC, non-metastatic PTC; mPTC, metastatic PTC; HS, Healthy subjects; NA, not available*.

Blood samples were collected in tubes treated with ethylenediaminetetraacetic acid (EDTA) before surgical operation and processed within 30 min post-collection. All specimens were centrifuged at 3,000 rpm for 10 min at 4°C to generate plasma. The supernatant was then fractioned into multiple aliquots for storage at −80°C until exosome and RNA isolation. All subjects provided informed consent before their plasma was drawn. The studies were conducted in accordance with the International Ethical Guidelines for Biomedical Research Involving Human Subjects, and the protocol was reviewed and approved by the Clinical Research Ethics Committee (CREC) of the Second Affiliated Hospital of Harbin Medical University.

### Isolation of Exosomes

Exosomes were isolated using the Exosome Precipitation Solution (EXOQ20A-1, SBI, Mountain View, CA) as previously mentioned (Wang et al., 2017). Briefly, 300 μL of plasma was thawed on ice and centrifuged at 3,000 g for 10 min to remove possible residual cell debris. After incubating with thromboplastin D (Thermo, Middletown, CA) at 37°C for 15 min and centrifuging, the 250 μL of supernatant was aspirated to a new tube and mixed with 65 μL of ExoQuick Solution. To digest free and lipoprotein-binding RNAs outside of small extracellular vesicles, RNaseA (Sigma, St. Louis, MO) was added to the mixture at a final concentration of 10 μg/mL. After keeping the mixture at 4°C overnight, murine RNase inhibitor (NEB, Ipswich, MA) was added at 150 units/mL before precipitation of exosomes by centrifuging at 1,500 g for 30 min. The exosome pellet was dissolved in 50 μL of phosphate-buffered saline (PBS) and subjected to RNA extraction immediately.

### Transmission Electron Microscopy (TEM)

The exosome suspension was diluted with PBS at a 1:1 ratio. Ten microliters of exosome re-suspension was dropped onto formvar-carbon-coated grids and blotted with filter paper after being sedated for 1 min. Then 10 μL of 3% phosphotungstic acid was dropped onto the exosome area for 1 min. After the excess staining buffer was removed with filter paper, the grid was left to air-dry for 5 min. Morphologic visualization of exosomes was carried out using a high-resolution transmission electron microscope (Hitachi HT7700, Japan) at 80 kV.

### Western Blot Analysis

PBS suspended exosomes were treated with RIPA lysis buffer (Beyotime, China) with a protease inhibitor (Roche, USA) and vortexed every 5–10 min for 30 min. Subsequently, the sample was spun at 14,000 g for 20 min to remove any debris and the supernatant was collected. Protein samples of 10 μg per lane were loaded onto 10% SDS-PAGE gels and transferred onto PVDF membranes (Millipore, USA) for 90 min. For immunodetection, the membranes were incubated with primary antibodies at 4°C overnight: CD63 (ab134045), CD81 (ab109201), TSG101 (ab125011), and Calnexin (ab22595) at 1:1,000 dilution. All primary antibodies were from Abcam, Cambridge, UK. The next morning the membranes were washed three times with TBS-T, 10 min each time. After incubation with a secondary antibody with gentle shaking at room temperature for 30–60 min, the membranes were washed with TBS-T, and the membrane was incubated with luminol substrate solution (Transgene, China) for 1 min. Signal of the target protein was collected with a Chemiluminescence imaging system (Proteinsimple, USA). All experiments were done at least five times.

### Flow Cytometry for Nanoparticle Analysis

Flow NanoAnalyzer model type N30 (NanoFCM Inc., Xiamen, China) was used to determine the size distribution and granular concentration of exosomes according to the manufacturer's instructions. Briefly, the isolated exosomes were diluted with PBS at 1:100 dilution. The Silica Nanospheres Cocktail (S16M-Exo, NanoFCM Inc.) was employed as size standard when constructing a calibration curve regarding particle sizes and side scattering intensities. Using this calibration curve, the side scattering intensity of every exosome was converted into the corresponding vesicle size.

### RNA Isolation

The miRNAs were extracted from plasma-isolated exosomes using the miRNeasy Micro Kit per the manufacturer's protocol (QIAGEN, Valencia, CA). The extracted RNA was eluted with 14 μL of RNase-free water. In the validation phase, the on-column binding RNA was treated with 30 units of DNase I for 15 min at room temperature before being eluted with H_2_O. The quality, yield, and distribution of miRNAs were analyzed using the Agilent 2100 bioanalyzer with Small RNA Chips (Agilent Technologies, Santa Clara, CA).

### Small RNA Library Preparation

Small RNA libraries were constructed as instructed by the NEBNext Multiplex Small RNA Library Prep Set for Illumina (NEB, Ipswich, MA, USA), with minor modifications. Ten nanograms of small RNA were used as the starting material. After sequential ligation of the Multiplex 3′ SR Adaptor, hybridization of the reverse transcription primer, and ligation of the Multiplex 5′ SR Adaptor, the RNA-Adaptor complex was reverse transcribed into cDNA. Then, the small RNA libraries were amplified, and the Illumina-compatible Index Primers were incorporated with 10 cycles of PCR amplification. The amplified libraries were resolved on a native 5% acrylamide gel (Bio-rad, Hercules, CA). DNA fragments corresponding to 140-160 bp (the size comprising the miRNA inserts plus the 3′ and 5′ adaptors) were recovered in 12 μL of elution buffer (QIAGEN) and determined with a high-sensitivity DNA chip on Agilent 2100 bioanalyzer. Absolute quantities of each indexed library were determined by real-time qPCR using the KAPA Library Quantification Kit according to the manufacturer's protocol. Ten microliters of the pooled libraries (consisting of 12 indexed libraries) at a final concentration of 2 nM were submitted for sequencing.

### Next-Generation Sequencing and Data Analysis

Next-generation sequencing was carried out on an Illumina HiSeq2000 platform by Novogen Inc. (Beijing, China). The clustering of the index-coded samples was performed on a cBot Cluster Generation System using TruSeq SR Cluster Kit v3-cBot-HS (Illumia). After cluster generation, the libraries were sequenced and 50-bp single-end reads were generated. The raw data (raw reads) extracted from FASTQ files were firstly processed through Perl and Python scripts. In this step, clean data (clean reads) were generated by removing reads containing ploy-N, with 5′ adapter contaminants, without 3′ adapter or the insert tag, containing ploy A or T or G or C and low-quality reads from raw data. Then, the clean data were aligned against human miRNA sequences from miRBase (Release 20) and NCBI human genome reference sequences (Release 103) using Bowtie (version 0.12.8). Differential expressed miRNAs between groups were determined by software DESeq2 using the read counts of each miRNA. To find novel miRNAs, the raw data was independently processed by a combined use of miRDeep 2 and miREvo. Predicted miRNAs with scores ≥2 were considered significant. To make every unique small RNA map to only one annotation, the data were processed with the following priority rule: known miRNA > rRNA > tRNA > snRNA > snoRNA > repeat > gene > NAT-siRNA > gene > novel miRNA > ta-siRNA.

### Quantitative Real-Time PCR (qRT-PCR)

To validate miRNAs identified by RNA sequencing, miScript SYBR Green PCR Kit (QIAGEN) was used to measure the expression levels of miR-16-2-3p, miR-34c-5p, miR-223-3p, miR-223-5p, miR-182-5p, miR-146b-5p, miR-101-3p, and miR-381-3p (MS00008813, MS00003332, MS00003871, MS00009184, MS00008855, MS00003542, MS00008372, and MIMAT0000736, respectively). MiR-30e-5p (MS00009401, Qiagen) was selected as an endogenous normalizer according to our previous study. For cDNA synthesis, a total of 4 ng of exosomal RNA (determined by an Agilent Small RNA Chip) from each sample was reverse transcribed using a QIAGEN miScript II RT kit with HighFlex buffer and a universal reverse transcription (RT) primer. Two microliters of each diluted cDNA product (1:40 in H_2_O) was used for 10-μL real-time PCR reactions containing 5 μL of QIAGEN SYBR green Master Mix, 1 μL of universal downstream primer, and 1 μL of miRNA specific upstream primer. Then, the real-time PCR was carried out on a LightCycler® 480 System (Roche Diagnostics, Mannheim, Germany) and the reactions were set as follows: 95°C for 15 min, 40 cycles of 94°C for 15 s, 55°C for 30 s, and 72°C for 30 s. All experiments were independently repeated three times to remove any outliers.

### Statistical Analysis

Relative miRNA expressions were calculated using the ΔC_T_ method (ΔC_T_ = C_TmiR_ – C_Treference_). Data were presented as the mean ± SD. For the multivariate prediction model, we calculated the risk score by a linear combination of the Ct values from real-time PCR for the selected miRNAs and/or clinical variables. We used GraphPad Prism version 6.0 (GraphPad Software, La Jolla, CA, USA) and SPSS software version 20.0 (IBM Corp., Armonk, NY, USA) for statistical analyses. An unpaired Student's *t*-test was performed to analyze the differences in miRNA expression between the two groups. A two-sided *p* < 0.05 was considered statistically significant. We applied a receiver operating characteristic (ROC) curve to determine the discrimination efficiencies of the exosomal miRNA, and logistic regression adjusted for sex and age to determine the diagnostic priority of the combination of exosomal miRNAs. The area under the curve (AUC) was calculated to assess the diagnostic power of the predictors. AUC can be used as an accurate measurement of the diagnostic marker; the larger the AUC, the better the prediction model. AUC = 0.5 indicates no predictive power, whereas AUC = 1 represents perfect predictive performance.

## Results

### Exosomes and Exosomal miRNA in a Limited Volume of Plasma Are Qualified to Establish Small RNA Libraries

**Figure 1:**
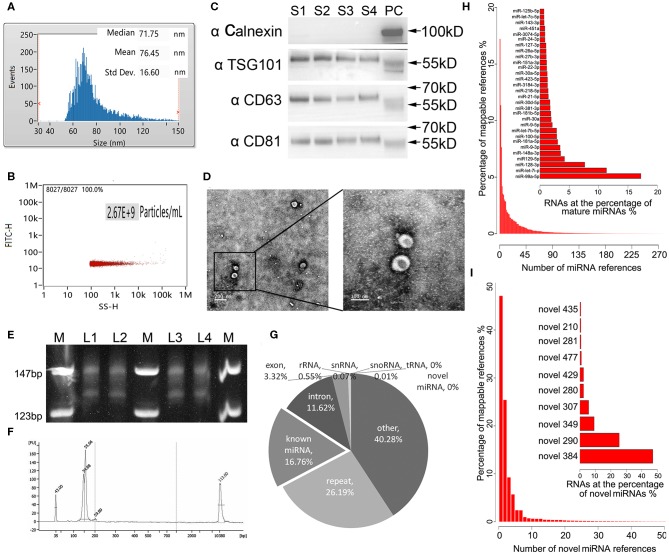
Technical pipeline for identification of plasma exosomal miRNAs by next-generation sequencing. **(A)** Representative size distribution of exosomes isolated from plasma determined using nano-flow cytometry. **(B)** Representative particle concentration of exosomes isolated from plasma determined by nano-flow cytometry. **(C)** Western blot showing the expression level of the typical exosome markers Calnexin (negative in exosomes), TSG-101, CD63, and CD81(positive in exosomes) in the four randomly selected exosomal samples from each group. S1–4, sample 1–4. PC, positive control using whole cell lysate prepared from HEK 293 cells. **(D)** Transmission electron microscopy picture showing the morphology of the exosomes. Scale bar on the left represents 200 nm, on the right 100 nm. **(E)** Polyacrylamide gel electrophoresis (PAGE) analysis for the established small RNA libraries. DNA ladders are shown in the 1st, 4th, and 7th lanes of the panel, while the DNA bands of prepared libraries are shown between the marker lanes. The anticipated size of the RNA sequencing constructs is ~150 bp. The DNA bands corresponding to 130 bp are the adaptor dimmers. M, DNA ladder. L1–4, library 1–4. **(F)** High-sensitivity DNA chip run on Agilent 2100 bioanalyzer demonstrates the quality and quantity of the gel-recovered library. **(G)** Illustrated RNA species and their abundance in the raw reads of exosomal RNA. Mappable reads are the sequences that are mapped to known human RNAs. lncRNA, long noncoding RNA; miRNA, microRNA; piRNA, piwi-interacting RNA; rRNA, ribosomal RNA; snRNA, small nuclear RNA; snoRNA, small nucleolar RNA; tRNA, transfer RNA. **(H)** Top 30 known miRNAs with the highest expression levels in exosomal miRNA libraries. **(I)** Top 10 novel miRNAs predicted by miRDeep2 and miREvo with the highest expression levels in exosomal miRNA libraries.

Exosomes as intraluminal vesicles inside multivesicular bodies range from 30 to 100 nm in size. Currently, available methods for isolation of this kind of extracellular vesicle from body fluids include ultracentrifugation, density gradient separation, chromatography, and immunoaffinity capture techniques. As in the past, we used ExoQuick Exosome Precipitation Solution to isolate exosomes from plasma for RNA-seq in the present study (Xu et al., 2016; Li et al., 2017). To confirm that the purified materials mainly consisted of exosomes, nano flow analysis was applied. The representative isolated exosomes from the patients' plasma samples displayed mainly around 71.75 nm size (76.45 ± 16.60 nm, Figure 1A), with concentration of 2.67 × 10^9^ particles/mL (Figure 1B. Refer to Supplementary Figure 1 for the detections of the four samples randomly selected from each group). Meanwhile, the common exosome markers CD81, CD63, and TSG-101, but not Calnexin, were present in all samples randomly selected from each group, as evidenced in the western blotting results (Figure 1C. The whole membrane picture has been provided in Supplementary Figure 2). Furthermore, TEM not only clearly showed the sizes of the vesicles with a diameter of ~100 nm, but also revealed the exosomes with a classic morphology of extracellular vehicle (Figure 1D). Using exosomes derived from 250 μL of plasma, miRNA yields were from 8 to 12 ng, with a median of 10.4 ng, which was slightly lower than our previous isolation under the same conditions, where a range of 10–15 ng exosomal miRNA was obtained. Evidenced by the explicit library DNA bands at 150 bp, Figure 1E demonstrates that exosomal RNA isolated using our optimized method can generate high-quality RNA-Seq libraries using NEBNext multiplex small RNA library preparation kit (NEB) with 10 ng of exosomal RNA isolated from a plasmatic specimen. The size of library fragments was further validated by a high-sensitivity DNA chip run on an Agilent Bioanalyzer 2100 platform (Figure 1F).

For the 24 RNA libraries, the raw sequencing data have been deposited in the GEO database (accession number: GSE130512), and the main sequencing parameters, including clean data and reads for each sample, are summarized in Supplementary Table 1. We received an average of 8.5 M mappable reads per library. These mappable reads aligned to RNA references were abundant in miRNAs (16.76%), intron (11.62%), exon (3.32%), repeat RNA (26.19%), and other RNA (40.28%), while infrequent in rRNA (0.55%), snRNA 0.07%, snoRNA (0.01%), tRNA (0%), and novel miRNA (0%) (Figure 1G). Consistent with our previous study, miR-99a-5p was the top abundant known miRNA in our libraries (>15%) (Yuan et al., 2016). Apart from the known mature miRNA, we also found some putative miRNAs with miRDeep2 in our libraries, where novel_384 accounted for the most abundant one. The top 30 abundant known mature miRNAs and the top 10 abundant novel miRNAs are shown in Figures 1H,I, respectively.

### Plasma Exosomal miRNA Signatures Discriminated Between Benign Thyroid Nodules and PTC in the Discovery Cohort

To discover diagnostic candidates, miRNA profiles derived from the plasma exosomes of the PTC and the NG patients were compared for statistical significance using DESeq2. We identified 14 miRNAs that were able to discriminate the NG group from the non-metastatic PTC (nmPTC) group (Table 2). We further combined metastatic PTC (mPTC) into the nmPTC and compared the NG with the total PTC for differentially expressed miRNAs (Table 3). Among the 16 candidates identified, 12 miRNAs were also differentially expressed between the NG and the nmPTC groups (Tables 2, 3, in bold). We also compared the nmPTC and mPTC groups and found 18 differentially expressed miRNAs (Table 4). None of the metastatic-associated miRNAs simultaneously differentiated the NG from the PTC patients regardless of metastatic status. By comprehensively considering the expression levels, significance of the differences, and fold changes between two groups, we selected a final five candidates (miR-16-2-3p, miR-34c-5p, miR-223-3p, miR-223-5p, and miR-101-3p) that showed differential expression between the NG and nmPTC group and between the NG and PTC groups for subsequent quantitative real-time PCR (qRT-PCR) validation. Meanwhile, miR-182-5p and miR-381-3p, the top 2 significant miRNAs between nmPTC and mPTC, were also included in the validation. MiR-146-5p, though less significant between NG and nmPTC or between the NG and PTC groups, was also included because previous studies demonstrated its diagnostic potential for PTC (Yu et al., 2012; Hardin et al., 2014; Deng et al., 2015; Lima et al., 2016; Jia et al., 2019). For the ease of downstream qRT-PCR using commercially available primers and possible clinical application in the future, putative miRNA novel_290 was excluded in the validation stage. We used miR-30e-5p as an endogenous normalizer as previously described.

**Table 2:** DESeq2 derived differential expressed exosomal miRNAs in the NG and the nmPTC patients.

**miRNA**	**Copy number in NG**	**Copy number in nmPTC**	Fold change^*^	log2 Fold change^*^	***P*-value**
**hsa-miR-223-3p**	20.7130	40.6152	1.9609	0.9715	0.0002
**hsa-miR-433-3p**	4273.3690	5110.1350	1.1958	0.2580	0.0115
**hsa-miR-425-5p**	429.4225	535.0175	1.2459	0.3172	0.0146
**hsa-miR-34c-5p**	685.0961	483.8378	0.7062	−0.5018	0.0200
**hsa-miR-132-5p**	820.8775	1041.2550	1.2685	0.3431	0.0248
**hsa-miR-9-5p**	32221.2200	25591.5000	0.7942	−0.3323	0.0259
hsa-miR-532-5p	43.0588	60.9037	1.4144	0.5002	0.0264
**hsa-miR-223-5p**	42.1606	65.5428	1.5546	0.6365	0.0271
hsa-miR-142-5p	171.6290	207.7171	1.2103	0.2753	0.0354
**hsa-miR-101-3p**	8633.9770	5837.4340	0.6761	−0.5647	0.0360
**hsa-miR-16-2-3p**	26.1871	39.0585	1.4915	0.5768	0.0408
**hsa-miR-1307-3p**	75.6830	102.0072	1.3478	0.4306	0.0425
**hsa-miR-26a-5p**	12521.5100	14592.2700	1.1654	0.2208	0.0440
**hsa-miR-146b-5p**	55.2837	81.9272	1.4819	0.5675	0.0477

* *The nmPTC compared with the NG*.

**Table 3:** DESeq2 derived differential expressed exosomal miRNAs in the NG and the PTC patients.

**miRNA**	**Copy number in NG**	**Copy number in PTC**	Fold change^*^	log2 Fold change^*^	***P*-value**
**hsa-miR-223-3p**	20.8523	42.9369	2.0591	1.0420	0.0001
**hsa-miR-433-3p**	4299.2820	5231.5230	1.2168	0.2831	0.0014
**hsa-miR-223-5p**	42.4282	73.2121	1.7256	0.7871	0.0087
**hsa-miR-34c-5p**	689.8310	496.3414	0.7195	−0.4749	0.0099
**hsa-miR-9-5p**	32429.2200	25864.9300	0.7976	−0.3263	0.0103
hsa-miR-320a	21450.4300	28130.3200	1.3114	0.3911	0.0130
**hsa-miR-16-2-3p**	26.3402	51.6409	1.9605	0.9712	0.0169
**hsa-miR-26a-5p**	12597.0100	14956.2000	1.1873	0.2477	0.0181
**hsa-miR-146b-5p**	55.6671	95.1267	1.7088	0.7730	0.0182
**hsa-miR-101-3p**	8696.7600	6040.6510	0.6946	−0.5258	0.0219
hsa-miR-655-3p	136.2349	104.4800	0.7669	−0.3829	0.0335
**hsa-miR-1307-3p**	76.0886	103.9587	1.3663	0.4503	0.0336
hsa-miR-133a-3p	63.2028	50.8078	0.8039	−0.3149	0.0394
**hsa-miR-425-5p**	431.9370	509.6795	1.1800	0.2388	0.0424
hsa-miR-455-5p	491.6646	571.0372	1.1614	0.2159	0.0425
**hsa-miR-132-5p**	825.8046	1005.1270	1.2171	0.2835	0.0478

* *The PTC compared with the NG*.

**Table 4:** DESeq2 derived differential expressed exosomal miRNAs in the mPTC and the nmPTC patients.

**miRNA**	**Copy number in nmPTC**	**Copy number in mPTC**	Fold change^*^	log2 Fold change^*^	***P*-value**
hsa-miR-182-5p	45.8480	89.5550	1.9533	0.9659	0.0050
hsa-miR-381-3p	29175.3000	14174.5300	0.4858	−1.0414	0.0063
hsa-miR-28-5p	98.9788	72.3537	0.7310	−0.4521	0.0085
hsa-miR-323a-5p	32.6358	14.3850	0.4408	−1.1819	0.0176
hsa-miR-128-3p	121166.8000	60780.9100	0.5016	−0.9953	0.0193
novel_290	53.5746	20.1747	0.3766	−1.4090	0.0202
hsa-miR-363-3p	255.2171	345.8804	1.3552	0.4385	0.0214
hsa-miR-543	2346.4710	1065.5250	0.4541	−1.1389	0.0229
hsa-miR-1912	24.7391	9.8238	0.3971	−1.3324	0.0231
hsa-miR-409-5p	52.7586	35.2117	0.6674	−0.5834	0.0238
hsa-miR-126-3p	4299.4550	6058.0680	1.4090	0.4947	0.0300
hsa-miR-32-5p	71.3650	98.5245	1.3806	0.4653	0.0347
hsa-miR-490-3p	278.2609	215.3766	0.7740	−0.3696	0.0356
hsa-miR-885-3p	111.5172	72.7431	0.6523	−0.6164	0.0360
hsa-miR-139-5p	2455.0090	1590.2210	0.6477	−0.6265	0.0398
hsa-miR-542-3p	40.5135	56.4215	1.3927	0.4778	0.0452
hsa-miR-151a-5p	108.6865	81.2446	0.7475	−0.4198	0.0465
hsa-miR-26b-5p	218.5468	328.8495	1.5047	0.5895	0.0487

* *The mPTC compared with the nmPTC*.

### Validation of Selected Exosomal miRNAs by qRT-PCR

**Figure 2:**
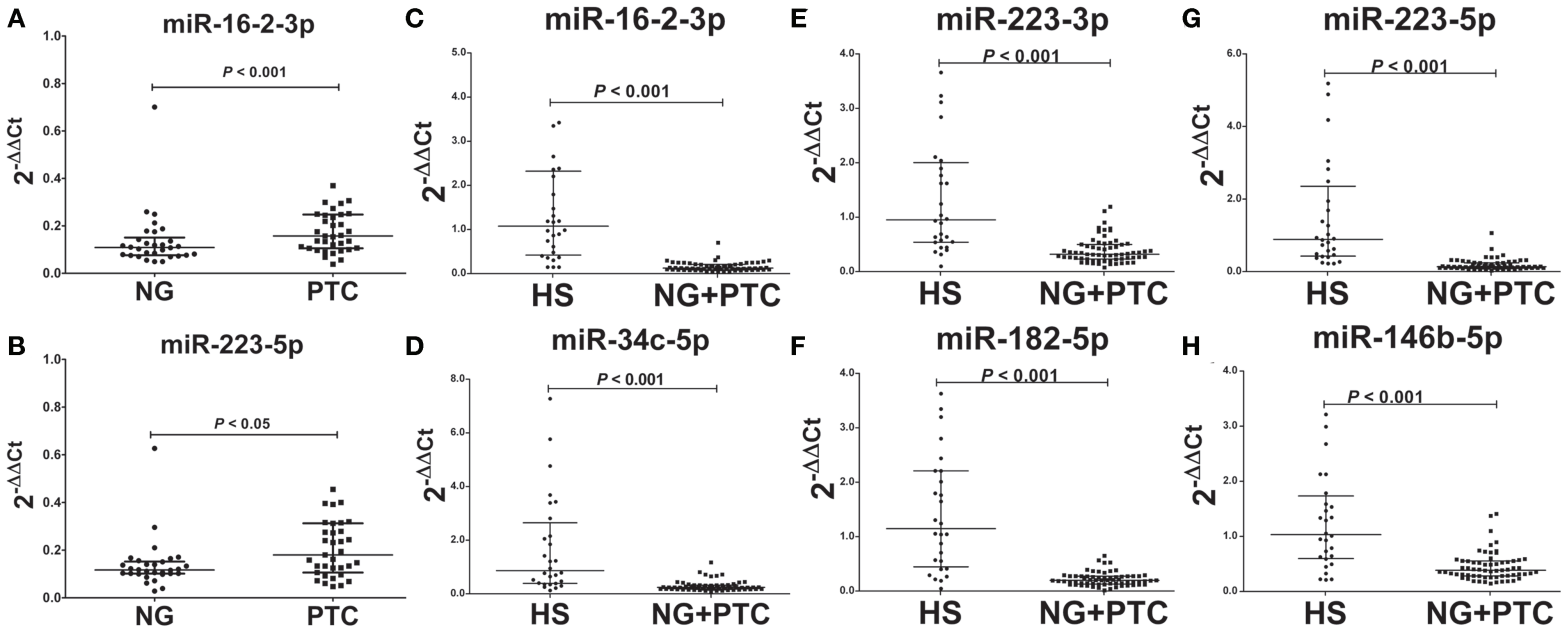
Expression levels of the selected plasma exosomal miRNA candidates in the validation set. Plasma exosomal miRNA was isolated from 30 benign thyroid nodules, 35 PTC cases, and 31 healthy controls. A total of 4 ng of miRNA was reverse transcribed using a QIAGEN miScript II RT kit. After 1:10 dilution, 2 μL of cDNA template was used for quantification by real-time PCR. Plasma levels of exosomal **(A)** miR-16-2-3p (*p* < 0.001) and **(B)** miR-223-5p (*p* < 0.05) were significantly higher in PTC compared with benign thyroid nodules. Levels of exosomal **(C)** miR-16-2-3p (*p* < 0.001), **(D)** miR-223-5p (*p* < 0.001), **(E)** miR-34c-5p (*p* < 0.001), **(F)** miR-182-5p (*p* < 0.001), **(G)** miR-223-3p (*p* < 0.001), and **(H)** miR-146b-5p (*p* < 0.001) were significantly lower in benign thyroid nodules and PTC compared with healthy subjects (HS). MiR-30e-5p was used as an internal control. The relative difference between the groups was calculated as 2^−ΔΔCt^. Horizontal lines: median with interquartile range.

In our validation cohort, we tested another 35 PTC patients (19 mPTC and 16 nmPTC) and 30 NG patients by qRT-PCR for expression differences in miR-16-2-3p, miR-34c-5p, miR-223-3p, miR-182-5p, miR-223-5p, miR-101-3p, miR-381-3p, and miR-146b-5p. As shown in Figures 2A,B, miR-16-2-3p and miR-223-5p were upregulated in PTC (3.10-, 1.62-fold, respectively, *p* < 0.05) compared with the NG groups, which were consistent with their expressive patterns in the discovery stage (1.96-, 2.05-fold, respectively, *p* < 0.05, Table 3). For metastasis-related miRNAs (miR-182-5p and miR-381-3p), we failed to find statistical significance between mPTC and nmPTC in the validation cohorts.

To determine the diagnostic value of these validated biomarkers in routine physical examination, 31 healthy individuals (HS group) were included in the validation stage, and the signatures of the eight plasma exosomal miRNAs were further examined. Compared to the HS group, the patient groups (PTC and NG patients) showed significantly reduced levels of miR-16-2-3p (0.17-fold, *p* < 0.05), miR-34c-5p (0.17-fold, *p* < 0.05), miR-223-3p (0.27-fold, *p* < 0.05), miR-182-5p (0.13-fold, *p* < 0.05), miR-223-5p (0.11-fold, *p* < 0.05), and miR-146b-5p (0.37-fold, *p* < 0.05) (Figures 2C–H), whereas the levels of miR-101-3p and miR-182-5p remained similar (*p* > 0.05).

### Diagnostic Performance of Exosomal miRNA Panels to Detect Malignant Nodules

**Figure 3:**
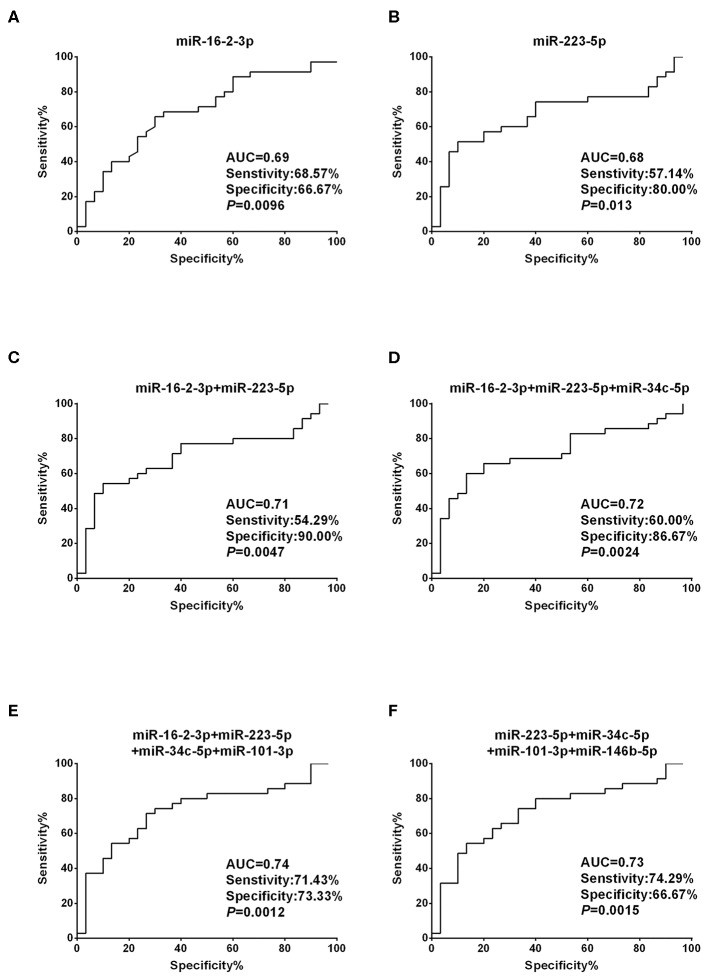
Diagnostic value based on plasma exosomal miR-16-2-3p and miR-223-5p in PTC. **(A,B)** ROC curve analyses of **(A)** miR-16-2-3p and **(B)** miR-223-5p in the discrimination of patients with PTC from patients with benign nodules (NG). **(C–F)** ROC curve analyses of combined miRNAs to distinguish patients with PTC from patients with benign nodules. The AUCs with specificity and sensitivity generated from **(C)** the 2 miRNAs (miR-16-2-3p and miR-223-5p), **(D)** 3 miRNAs (miR-16-2-3p, miR-223-5p, miR-34c-5p), **(E)** 4 miRNAs (miR-16-2-3p, miR-223-5p, miR-34c-5p, miR-101-3p), and **(F)** 4 miRNAs (miR-223-5p, miR-34c-5p, miR-101-3p,miR-146b-5p) in PTC vs. NG are shown.

To assess the diagnostic performance of the selected miRNA candidates in discriminating PTC from benign thyroid nodules, ROC curve analyses were carried out using the data generated in the validation set. The AUCs were 0.69 (sensitivity: 68.57% and specificity: 66.67%) and 0.68 (sensitivity: 57.14% and specificity: 80%) for miR-16-2-3p and miR-223-5p, respectively (Figures 3A,B, Supplementary Table 2). None of the other six miRNA candidates demonstrated significantly larger AUCs than miR-16-2-3p and miR-223-5p (Supplementary Table 2). To address the heterogeneity issue, we combined multiple miRNAs to calculate multivariate-based predictive models. When combining miR-16-2-3p with miR-223-5p, the AUC was increased to 0.71 with sensitivity at 54.29% and specificity at 90.00% (Figure 3C). When miR-34c-5p was further incorporated into this model, the AUC was elevated to 0.72 with higher efficiency (sensitivity: 60.00% and specificity: 86.67%) (Figure 3D). By adding miR-101-3p to generate a 4-miRNA model, the AUC was further increased to 0.74 with sensitivity at 71.43% and specificity at 73.33% (Figure 3E). However, the AUC was reduced when miR-146b-5p instead of miR-16-2-3p was included in the panel (AUC = 0.73, sensitivity: 74.29% and specificity: 66.67%, Figure 3F). Collectively, the 4-miRNA panel (miR-16-2-3p, miR-223-5p, miR-34c-5p, and miR-101-3p) accounts for the most powerful biomarkers in discriminating NG from PTC (Supplementary Table 2).

**Figure 4:**
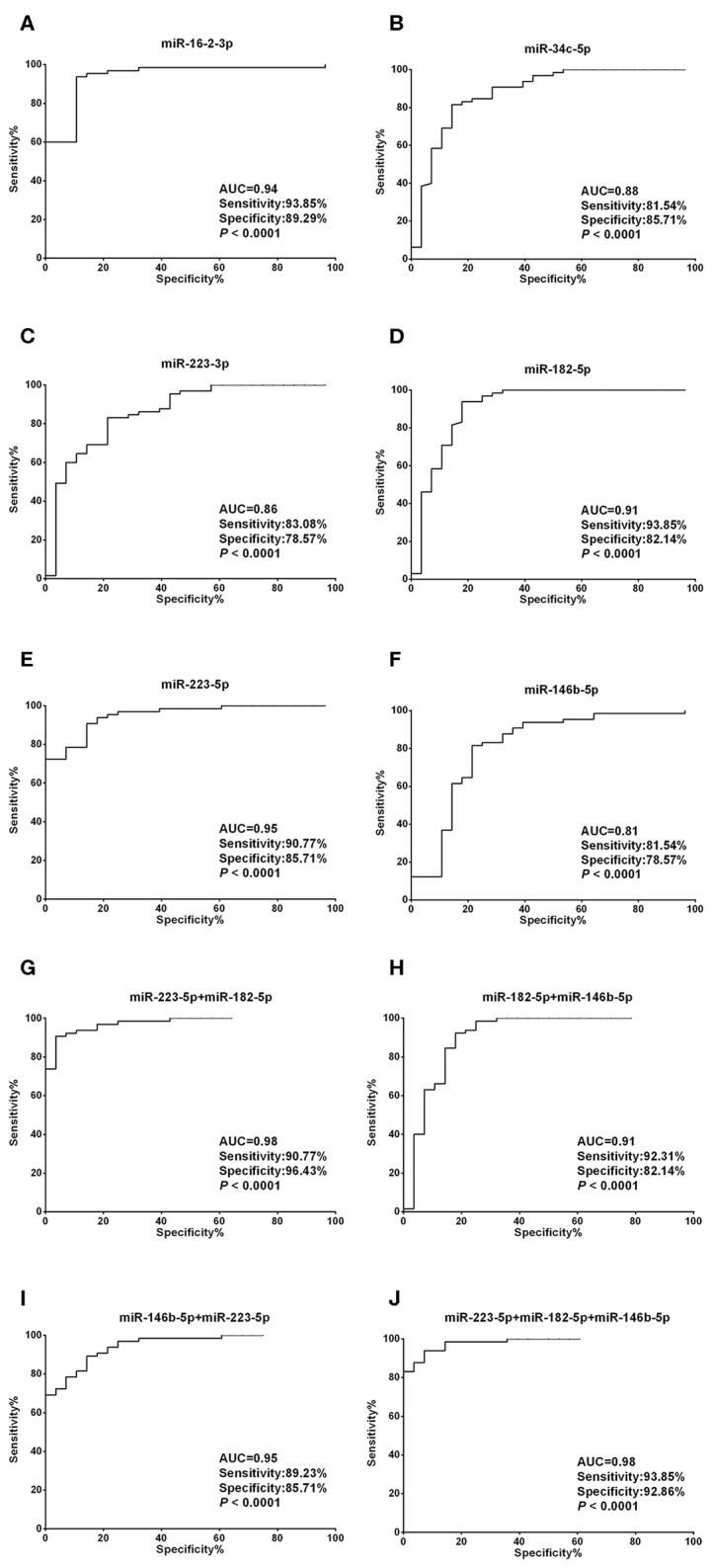
Diagnostic value of each plasma exosomal miRNA marker in thyroid nodules. **(A–F)** ROC curve analyses of ΔCt summation of **(A)** miR-16-2-3p, **(B)** miR-223-5p, **(C)** miR-34c-5p, **(D)** miR-223-3p, **(E)** miR-182-5p, and **(F)** miR-146b-5p as parameters to discriminate between thyroid nodules and healthy control cases. **(G–I)** Combining **(G)** miR-223-5p and miR-182-5p, **(H)** miR-182-5p and miR-146b-5p, **(I)** miR-146b-5p and miR-223-5p, created enhanced AUC values of 0.975, 0.907, 0.949, a sensitivity of 90.77, 92.31, 89.23%, and a specificity of 96.43, 82.14, and 85.71% in identifying thyroid nodules out of the validation set. **(J)** MiR-223-5p, miR-182-5p, and miR-146b-5p by qPCR ΔCt summation produces an AUC value of 0.982, a sensitivity of 93.85%, and a specificity of 92.86% in identifying thyroid nodules out of the validation set.

Unlike the moderate difference between thyroid cancer and nodular goiters, most miRNA candidates performed much better in discriminating thyroid nodules from healthy individuals. With the exception of miR-34c-5p, miR-223-3p, and miR-146b-5p, the AUCs were each above 0.90, with both sensitivity and specificity >80% for miR-16-2-3p, miR-223-5p, and miR-182-5p (Figures 4A–F, Supplementary Table 3). We also tested 2-miRNA models for their diagnostic power. The combination of miR-223-5p and miR-182-5p could discriminate nodular patients from healthy subjects with an AUC of 0.98 with 90.77% sensitivity and 96.43% specificity (Figure 4G), followed by miR-223-5p and miR-146b-5p for an AUC of 0.95 with 89.23% sensitivity and 85.71% specificity (Figure 4H), and miR-182-5p and miR-146b-5p for an AUC of 0.91 with 92.31% sensitivity and 82.14% specificity (Figure 4I). When the 3-miRNA panel was constructed, the combination of miR-223-5p, miR-182-5p, and miR-146b-5p showed the highest discriminative power with an AUC of 0.98 (sensitivity: 93.85% and specificity: 92.86%, Figure 4J) for differentiating the nodular patients from the healthy group (Supplementary Table 3).

### Expression and Possible Function of the Candidates in PTC

In order to detect to what extent the biomarkers are aberrant in PTC, we determined the expression patterns of the eight exosomal miRNAs in PTC tissues using online algorism ENCORI which performed analysis with the expression data downloaded from TCGA project via Genomic Data Commons Data Portal (http://starbase.sysu.edu.cn/) (Li et al., 2014). When compared to the 58 normal thyroid tissues, expression levels of all of the eight miRNAs were significantly altered in the 509 PTC tissues (Supplementary Table 4 and Supplementary Figure 3, *p* < 0.001 for all miRNAs). Similar to the trend in plasma exosomes, miR-146b-5p and miR-182-5p were increased, and miR-34c-5p, miR-101-3p, and miR-381-3p were decreased in thyroid cancer tissue. However, three miRNAs, miR-223-5p, miR-223-3p, and miR-381-3p, which were upregulated in plasma exosomes of the PTC patients, were found to be significantly downregulated in PTC tissues.

In terms of the possible function of the miRNA candidates, we used miRPath (http://www.microrna.gr/miRPathv3/) to compute the pathways the miRNAs are involved in Backes et al. (2017). As shown in the Supplementary Figure 4, sorted by GO (gene ontology) categories, the eight miRNAs clustered into two distinct groups. MiR-101-3p along with our metastasis-discriminating miRNAs, miR-182-5p and miR-381-3p, fell into one group, and the other five miRNAs into another. All of the eight miRNAs are targeting organelle-relevant pathways. Likewise, the KEGG (Kyoto Encyclopedia of Genes and Genomes) database also revealed two independent groups, where miR-182-5p and miR-381-3p were clustered into one and the other six into another (Supplementary Figure 5). Interestingly, the KEGG shows that miR-182-5p and miR-381-3p are mainly involved in cancer-related pathways, and targets of miR-146b-5p are significantly related with thyroid hormone synthesis.

## Discussion

Currently, FNA cytology, an invasive surgical pathological examination, is the gold standard diagnostic approach for ruling out benign thyroid nodules and PTC (Gonzalez-Gonzalez et al., 2011). However, nodules <1 cm are considered clinically insignificant (Haugen, 2017), which means that unless there exists extra thyroidal invasion, nodal metastases, or arguably, previous exposure to radiation or a family history of thyroid cancer, non-invasive biopsy should be the first priority. It is still urgent to develop sensitive and specific approaches as least invasive as possible for screening patients with asymptomatic thyroid nodules (Dean and Gharib, 2008) and diagnosing different histological sub-types of TC (Seethala et al., 2018). Recently, significant attention has been focused on the study of exosomal miRNAs for alternative cancer diagnosis in several types of cancer, such as colorectal cancer (Wang et al., 2017), prostate cancer, and hepatocellular carcinoma (Yuan et al., 2016). However, few studies have focused on plasma exosomal miRNA profiles to develop biomarkers for non-invasively detecting PTC, except one study that applied PCR panels to distinguish follicular TC from PTC through comparative assessment of plasma exosomal miR-21 and miR-181a-5p (Samsonov et al., 2016). In our present study, we performed RNA-seq analyses on plasma exosomal small RNA to find new known and putative miRNA biomarkers that are capable of distinguishing benign from malignant thyroid nodules. We also subsequently validated their diagnostic value using qRT-PCR.

Other than using the more stable fraction of blood, most of the current studies focused on whole plasma or serum to develop a miRNA biomarker for diagnosis and/or prognosis of PTC. One study determined serum miRNA expression profiles using a Solexa Sequencing Platform in patients with PTC, benign nodules, and healthy controls. They found that the circulating miRNA let-7e, miR-151, and miR-222 may serve as novel minimally invasive biomarkers for the diagnosis of PTC (Yu et al., 2012). Profiled with TaqMan Human MiRNA A Cards v2.0, serum miR-579, miR-95, miR-29b, and miR-190 were found to be differentially expressed in PTC (Cantara et al., 2014). Likewise, Exiqon miRCURY LNA miRNA Array in tissue or plasma suggested that circulating miR-222, miR-146b, miR-124-3p, miR-9-3p, and miR-196b were overexpressed in PTC and NG patients, although the potential of these miRNAs as diagnostic biomarkers for PTC surveillance was questionable because of the small size of the plasma sample (Lee et al., 2013; Yu et al., 2016). Additionally, genome-wide plasma miRNA expression profiles were also determined by Agilent Human miRNA Microarray, and the levels of plasma miR-25-3p and miR-451a may be valuable for the diagnosis of PTC (Li et al., 2015). Other investigations also revealed plasma miR-146b and miR-155 expression, showed that serum miR-222, miR-221, and miR-146b levels were higher in the PTC group, and were helpful to diagnose the benign and malignant lesions or PTC with and without lymph node metastasis (Lee et al., 2015; Zhang et al., 2017; Jahanbani et al., 2018). Among the diagnostic candidates, miR-146b-5p is the most intensively investigated biomarker. Numerous studies have demonstrated that miR-146b can be stably detected in serum or plasma and has the potential to be the novel diagnostic biomarker for diseases other than thyroid cancer (Bookland et al., 2018), while also playing a controversial role in the initiation and development of colorectal cancer, leukemia, and glioma (Li et al., 2013; Zhang et al., 2016; Yu et al., 2018; Jia et al., 2019). In line with the previous publication, plasma exosomal miR-146b-5p, along with another five newly defined miRNA biomarkers, were also eligible for early diagnosis of thyroid nodules in our study. To our knowledge, this is the first study to examine plasma exosomal miRNA expression in healthy individuals and benign and malignant thyroid nodules and to determine the diagnostic effectiveness of different combinations of a single biomarker. Though their diagnostic power is not as high as expected, the biomarkers we identified for now possess the highest specificity and sensitivity in non-invasive discrimination of thyroid nodules. Validation in large cohorts may effectively enhance their performance.

It is notable that the AUC value of the same miRNA for discriminating thyroid nodules from heathy ones is much higher than for malignant nodules from benign ones. In our opinion, the higher AUC is mainly owed to the stage of the medical condition. Fierce alterations in miRNA expression may occur when an organism transforms from healthy to unhealthy. After the thyrocytes have completed their initiating step to acquire enormous proliferative capacity, an integrated body response may be forced toward the baseline level by a homeostatic mechanism. Moreover, the relatively small sample size eliciting more unfair statistical varieties in our study may be another reason for the difference. Nevertheless, our diagnostic panel accounts for an ideal option in physical examination to distinguish nodules from healthy ones before the nodules can be detected with ultrasonography. Taken together, the existing evidence strongly supports the notion that circulating exosomal miRNA-based liquid biopsy is a promising option for clinical diagnosis of PTC.

Another interesting point is that levels of miR-16-2-3p and miR-223-5p are significantly higher in the plasma of patients with PTC than in benign thyroid nodules. However, the expression levels of these miRNAs were significantly lower in thyroid nodules than with healthy individuals. A possible interpretation of this phenomenon is that the expression of these two miRNAs might be a dynamic procedure during the process of carcinoma development. MiR-16-2-3p and miR-223-5p were initially downregulated as the benign nodule developed, but were significantly upregulated when the nodules transition into PTC due to the complicated oncogenetic events (Derwahl, 1996). Another possible reason is that the benign nodules and PTC are reciprocally independent thyroid diseases. Both miRNAs significantly decrease in the two medical conditions and are more downregulated in the benign nodule. Nevertheless, the underlying mechanisms in the regulation are worthy of further investigation.

Based on our results, the consistent expression patterns of the miRNAs in circulating exosomes of PTC patients and in PTC tissue, especially the highly abundant ones, such as miR-146b-5p, indicates the possible origin of the candidate biomarkers. When an abundant miRNA is strikingly modulated during tumorigenesis, either up or down regulated, it is rational to deduce that the fluctuation of the miRNA in circulation is a resultant aberrance affected by tumor cells. However, it is also of note that some miRNAs with low abundance, miR-16-2-3p for instance, are reversely expressed in circulating exosomes compared to PTC tissue. A similar contradiction was also noticed in our previous studies, suggesting the level of specific miRNA in circulating exosomes is an integrated response of the body during the course of tumorigenesis, and does not necessarily reflect its state in tumor tissue. However, miRNAs consistently expressed in circulation exosomes and tumor tissue deserve research priority. Further functional investigation concentrating on their interactive pathways revealed by GO and KEGG is strongly recommended.

In conclusion, this study identified two miRNAs (miR-16-2-3p and miR-223-5p) and six miRNAs (miR-16-2-3p, miR-34c-5p, miR-223-3p, miR-223-5p, miR-182-5p, and miR-146b-5p) as biomarkers for PTC and thyroid nodules. Upon further validation, plasma exosomal miRNA signatures should facilitate the earlier diagnosis of PTC. In the era of liquid biopsy, circulating exosomal miRNAs may offer an important resource for biomarker discovery and possess a great potential to assist in making clinical decisions, singly or in combination.

## Data Availability Statement

The raw sequencing data can be found in the GEO database (accession number: GSE130512).

## Ethics Statement

The studies involving human participants were reviewed and approved by Clinical Research Ethics Committee (CREC) of The Second Affiliated Hospital of Harbin Medical University. The patients/participants provided their written informed consent to participate in this study.

## Author Contributions

ML, XH, and SY conceived and designed the overall study. ML, SY, ST, LB, JC, and YG performed the experiments. SL, XZ, LD, LW, and YZ analyzed the data. ML and SY wrote the manuscript. XH revised the manuscript. All authors read and approved the final manuscript.

## Conflict of Interest

The authors declare that the research was conducted in the absence of any commercial or financial relationships that could be construed as a potential conflict of interest.
