# Supplementary material for: A Panel of Plasma Exosomal miRNAs as Potential Biomarkers for Differential Diagnosis of Thyroid Nodules
Source: Front Genet. 2020 May 19;11:449. doi: 10.3389/fgene.2020.00449 (PMC7248304; doi:10.3389/fgene.2020.00449)

Supplementary Material

**Supplementary Table S1**. Sequence read counts from RNA sequencing for the 24 libraries

|  | **Sample ID** | **Total Reads** | **known miRNA** | **rRNA** | **tRNA** | **snRNA** | **snoRNA** | **repeat** | **novel miRNA** | **exon:+** | **exon:-** | **intron:+** | **intron:-** | **other** |
| --- | --- | --- | --- | --- | --- | --- | --- | --- | --- | --- | --- | --- | --- | --- |
|  | NG1 | 2653679 | 774569 | 11938 | 3 | 2363 | 245 | 401436 | 77 | 99736 | 20996 | 144036 | 128036 | 1070244 |
|  | NG2 | 5711311 | 808900 | 25840 | 33 | 3512 | 567 | 1431707 | 139 | 129559 | 16972 | 496924 | 242428 | 2554730 |
|  | NG3 | 12577184 | 2404151 | 60660 | 55 | 9748 | 1518 | 2888034 | 254 | 398971 | 52001 | 1166254 | 696132 | 4899406 |
|  | NG4 | 4633238 | 600452 | 27692 | 53 | 4464 | 463 | 1085436 | 151 | 114737 | 15136 | 473756 | 240733 | 2070165 |
|  | NG5 | 7403264 | 1186256 | 33460 | 31 | 4075 | 753 | 1952553 | 166 | 142917 | 24280 | 669497 | 276351 | 3112925 |
|  | NG6 | 12122847 | 1949454 | 50490 | 54 | 6903 | 1204 | 3978020 | 174 | 278508 | 34344 | 1079941 | 389416 | 4354339 |
|  | NG7 | 15016782 | 3785576 | 220925 | 78 | 12537 | 2489 | 3472763 | 344 | 740517 | 82637 | 814240 | 602576 | 5282100 |
|  | NG8 | 10737196 | 1927592 | 46518 | 49 | 7680 | 1334 | 2925601 | 253 | 205740 | 41256 | 1012346 | 440308 | 4128519 |
|  | nmPTC1 | 10629829 | 1064438 | 49429 | 64 | 6453 | 843 | 3380452 | 143 | 285738 | 29265 | 962203 | 368641 | 4482160 |
|  | nmPTC2 | 11424609 | 1638929 | 60811 | 84 | 7271 | 1185 | 3608450 | 142 | 375803 | 36366 | 1105686 | 407757 | 4182125 |
|  | nmPTC3 | 5487740 | 727208 | 26301 | 26 | 3376 | 523 | 1471881 | 113 | 162946 | 15543 | 485010 | 238960 | 2355853 |
|  | nmPTC4 | 2356627 | 762624 | 7130 | 9 | 1484 | 214 | 388194 | 68 | 82638 | 18924 | 91727 | 80650 | 922965 |
|  | nmPTC5 | 10154035 | 1353190 | 56811 | 111 | 7047 | 833 | 2506659 | 231 | 228689 | 28122 | 935670 | 381810 | 4654862 |
|  | nmPTC6 | 9038000 | 1481914 | 43733 | 21 | 4045 | 746 | 2341790 | 204 | 314314 | 37278 | 251585 | 243903 | 4318467 |
|  | nmPTC7 | 5708101 | 1504967 | 23916 | 14 | 4794 | 484 | 1027778 | 159 | 326512 | 38068 | 276709 | 171810 | 2332890 |
|  | nmPTC8 | 7709346 | 2097780 | 47289 | 27 | 5929 | 750 | 1211473 | 239 | 386218 | 50125 | 372584 | 296243 | 3240689 |
|  | mPTC1 | 10908680 | 1059831 | 42956 | 54 | 5521 | 879 | 3299249 | 228 | 450313 | 34561 | 910638 | 368909 | 4735541 |
|  | mPTC2 | 10554449 | 2046953 | 43521 | 32 | 7456 | 1117 | 2854761 | 211 | 223311 | 36249 | 896622 | 414492 | 4029724 |
|  | mPTC3 | 7798301 | 925108 | 35834 | 44 | 5076 | 688 | 1986593 | 135 | 203113 | 19894 | 652783 | 253464 | 3715569 |
|  | mPTC4 | 7300785 | 1308325 | 36626 | 68 | 5359 | 805 | 1896964 | 156 | 202352 | 24971 | 667462 | 294463 | 2863234 |
|  | mPTC5 | 9989543 | 1532615 | 46974 | 72 | 9362 | 1158 | 2105404 | 495 | 261438 | 35418 | 965658 | 415572 | 4615377 |
|  | mPTC6 | 12645812 | 1825373 | 49040 | 62 | 8371 | 1073 | 3684240 | 249 | 217898 | 33523 | 973679 | 486045 | 5366259 |
|  | mPTC7 | 4184968 | 583137 | 20896 | 36 | 2763 | 414 | 1088562 | 69 | 126241 | 12127 | 357529 | 150280 | 1842914 |
|  | mPTC8 | 7298823 | 1304651 | 28639 | 46 | 3982 | 1064 | 1726280 | 187 | 222841 | 25403 | 595953 | 332809 | 3056968 |
| Average | | 8501881 | 1443916 | 45726 | 47 | 58157 | 890 | 2196428 | 191 | 257544 | 31811 | 681604 | 330075 | 3507834 |
| NG: Benign nodular goiters; nmPTC: non-metastatic PTC; mPTC: metastatic PTC; +: sense strand; -: antisense strand | | | | | | | | | | | | | | |

**Supplementary Table S2**. The performance of the plasma exosomal miRNAs in discrimination of the thyroid nodule from the thyroid cancer patients

| **miRNAs** | **AUC** | **Sensitivity%** | **95% CI** | **Specificity%** | **95% CI** | ***P* value** | **Cutoff** |
| --- | --- | --- | --- | --- | --- | --- | --- |
| miR-16-2-3p | 0.6876 | 68.57 | 50.71% - 83.15% | 66.67 | 47.19% - 82.71% | 0.009561 | > 0.1200 |
| miR-223-5p | 0.679 | 57.14 | 39.35% - 73.68% | 80 | 61.43% - 92.29% | 0.0134 | > 0.1573 |
| miR-16-2-3p+miR-223-5p | 0.7048 | 54.29 | 36.65% - 71.17% | 90 | 73.47% - 97.89% | 0.004685 | < 0.9223 |
| miR-223-5p+miR-34c-5p+miR-16-2-3p | 0.72 | 60 | 42.11% - 76.13% | 86.67 | 69.28% - 96.24% | 0.00238 | < 0.9314 |
| miR-223-5p+miR-34c-5p+miR101-3p+miR146b-5p | 0.7305 | 74.29 | 56.74% - 87.51% | 66.67 | 47.19% to 82.71% | 0.001459 | < 0.9460 |
| miR-223-5p +miR-34c-5p+miR101-3p+miR-16-2-3 | 0.7352 | 71.43 | 53.70% - 85.36% | 73.33 | 54.11% - 87.72% | 0.001161 | < 0.9468 |

**Supplementary Table S3.** The performance of the plasma exosomal miRNAs in discrimination of the healthy individuals from the thyroid nodule and the thyroid cancer patients

| **miRNAs** | **AUC** | **Sensitivity%** | **95% CI** | **Specificity%** | **95% CI** | ***P* value** | **Cutoff** |
| --- | --- | --- | --- | --- | --- | --- | --- |
| miR-223-3p | 0.861 | 83.08 | 71.73% - 91.24% | 78.57 | 59.05% - 91.70% | < 0.0001 | < 0.5366 |
| miR-34c-5p | 0.8805 | 81.54 | 69.97% - 90.08% | 85.71 | 67.33% - 95.97% | < 0.0001 | < 0.3399 |
| miR-223-5p | 0.95 | 90.77 | 80.98% - 96.54% | 85.71 | 67.33% - 95.97% | < 0.0001 | < 0.3462 |
| miR-16-2-3p | 0.9385 | 93.85 | 84.99% - 98.30% | 89.29 | 71.77% - 97.73% | < 0.0001 | < 0.3006 |
| miR-146b-5p | 0.8121 | 81.54 | 69.97% - 90.08% | 78.57 | 59.05% - 91.70% | < 0.0001 | < 0.5895 |
| miR-182-5p | 0.9085 | 93.85 | 84.99% - 98.30% | 82.14 | 63.11% - 93.94% | < 0.0001 | < 0.3967 |
| miR-223-5p+miR-182-5p | 0.9753 | 90.77 | 80.98% - 96.54% | 96.43 | 81.65% - 99.91% | < 0.0001 | > 0.8550 |
| miR-182-5p+miR-146b-5p | 0.9066 | 92.31 | 82.95% - 97.46% | 82.14 | 63.11% - 93.94% | < 0.0001 | > 0.7585 |
| miR-146b-5p+miR-223-5p | 0.9489 | 89.23 | 79.06% - 95.56% | 85.71 | 67.33% - 95.97% | < 0.0001 | > 0.7892 |
| miR-223-5p+miR-182-5p+miR-146b-5p | 0.9819 | 93.85 | 84.99% - 98.30% | 92.86 | 76.50% - 99.12% | < 0.0001 | > 0.7698 |

**Supplementary Table S4.** Aberrant expression of the exosomal miRNA biomarkers in PTC tissues compared with normal thyroid tissues

| **miRNA** | **Cancer** | **Normal** | **Fold change** | **P value** | **FDR** |
| --- | --- | --- | --- | --- | --- |
| miR-146b-5p | 23934.17 | 578.46 | 41.38 | 4.9e-36 | 1.3e-33 |
| miR-16-2-3p | 7.01 | 13.91 | 0.50 | 4.8e-19 | 2.2e-17 |
| miR-223-3p | 117.97 | 233.18 | 0.51 | 1.8e-13 | 4.6e-12 |
| miR-381-3p | 12.59 | 22.05 | 0.57 | 2.5e-8 | 3.2e-7 |
| miR-223-5p | 1.59 | 3.29 | 0.48 | 3.9e-6 | 3.4e-5 |
| miR-34c-5p | 4.04 | 5.29 | 0.76 | 1.9e-5 | 0.00015 |
| miR-182-5p | 13944.25 | 8098.43 | 1.72 | 2.0e-5 | 0.00015 |
| miR-101-3p | 29159.96 | 34484.67 | 0.85 | 0.00051 | 0.0028 |

**Supplementary Figure S1.** Detection of the size and concentration of the exosomes obtained from 4 samples from each group

**
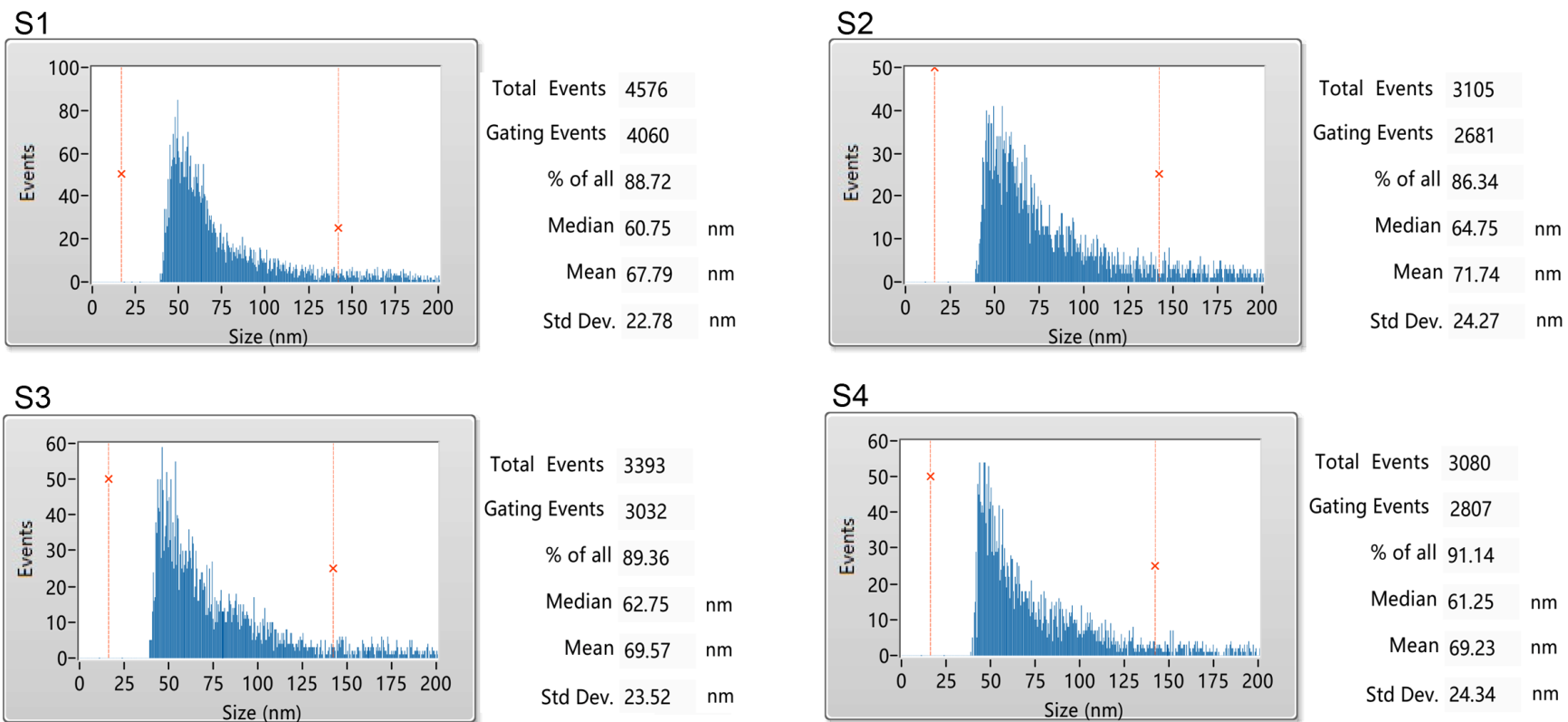
**

**Supplementary Figure S2.** Whole membrane picture about western blot toward 4 typical exosome markers

**
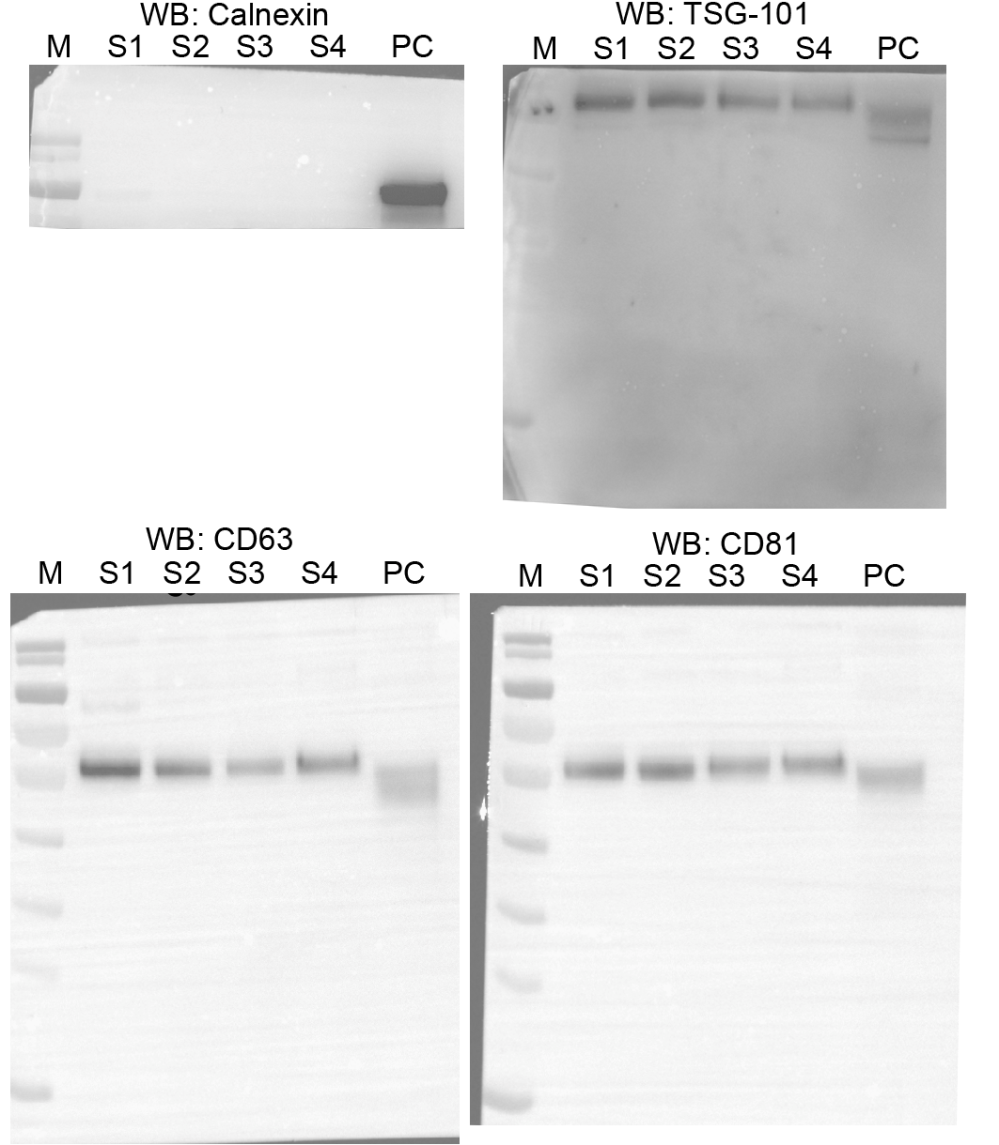
**

**
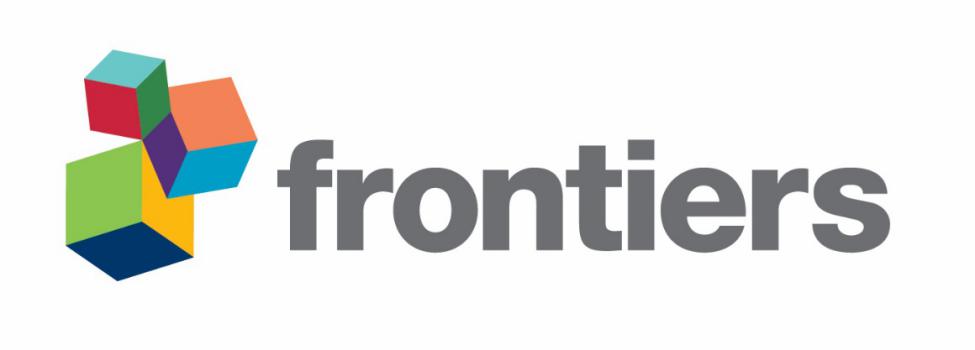
**

Supplementary Figure S3. Expression patterns of the diagnostic candidates in PTC tissues


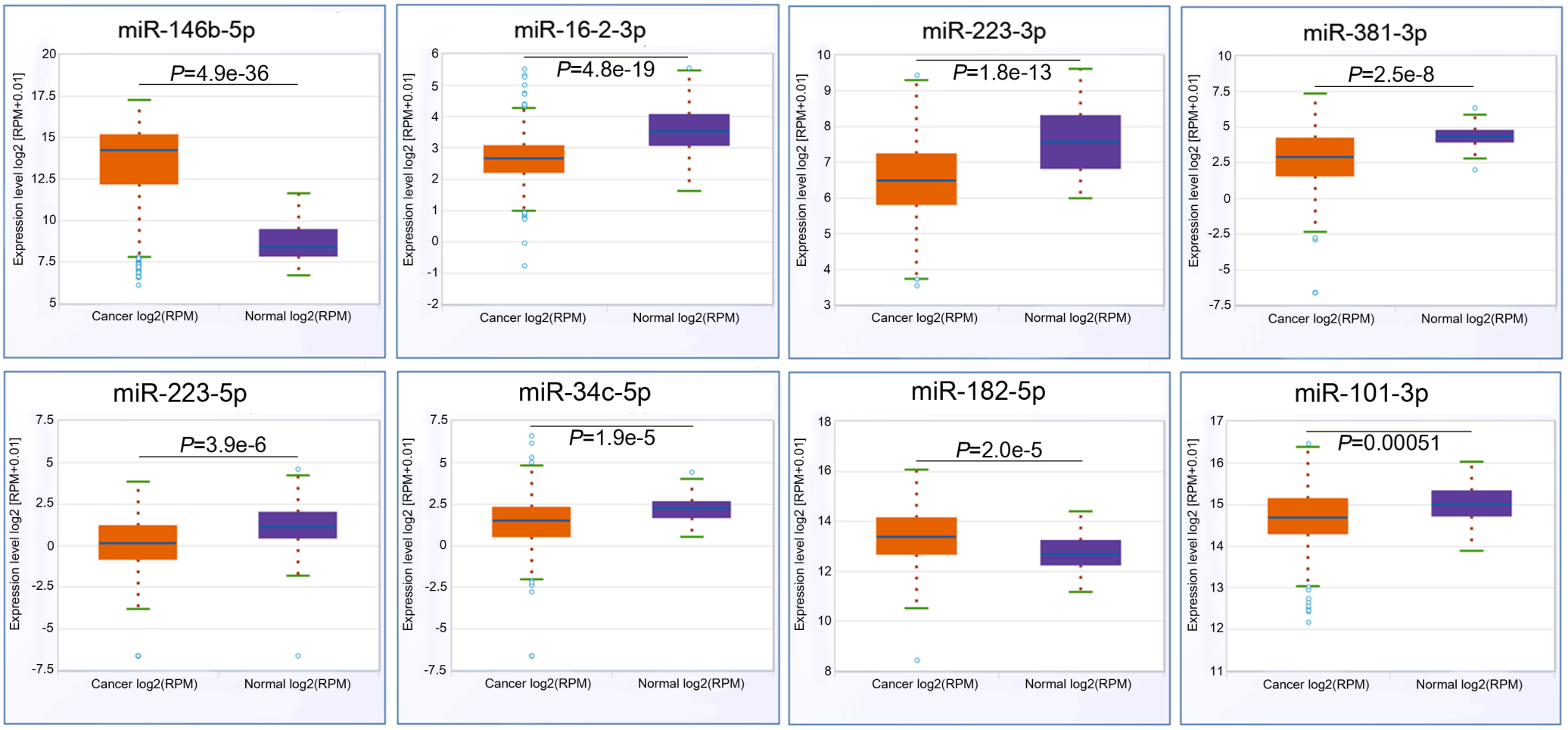


Supplementary Figure S4, Interactive heat maps showing the GO enriched pathways targeted by the miRNAs


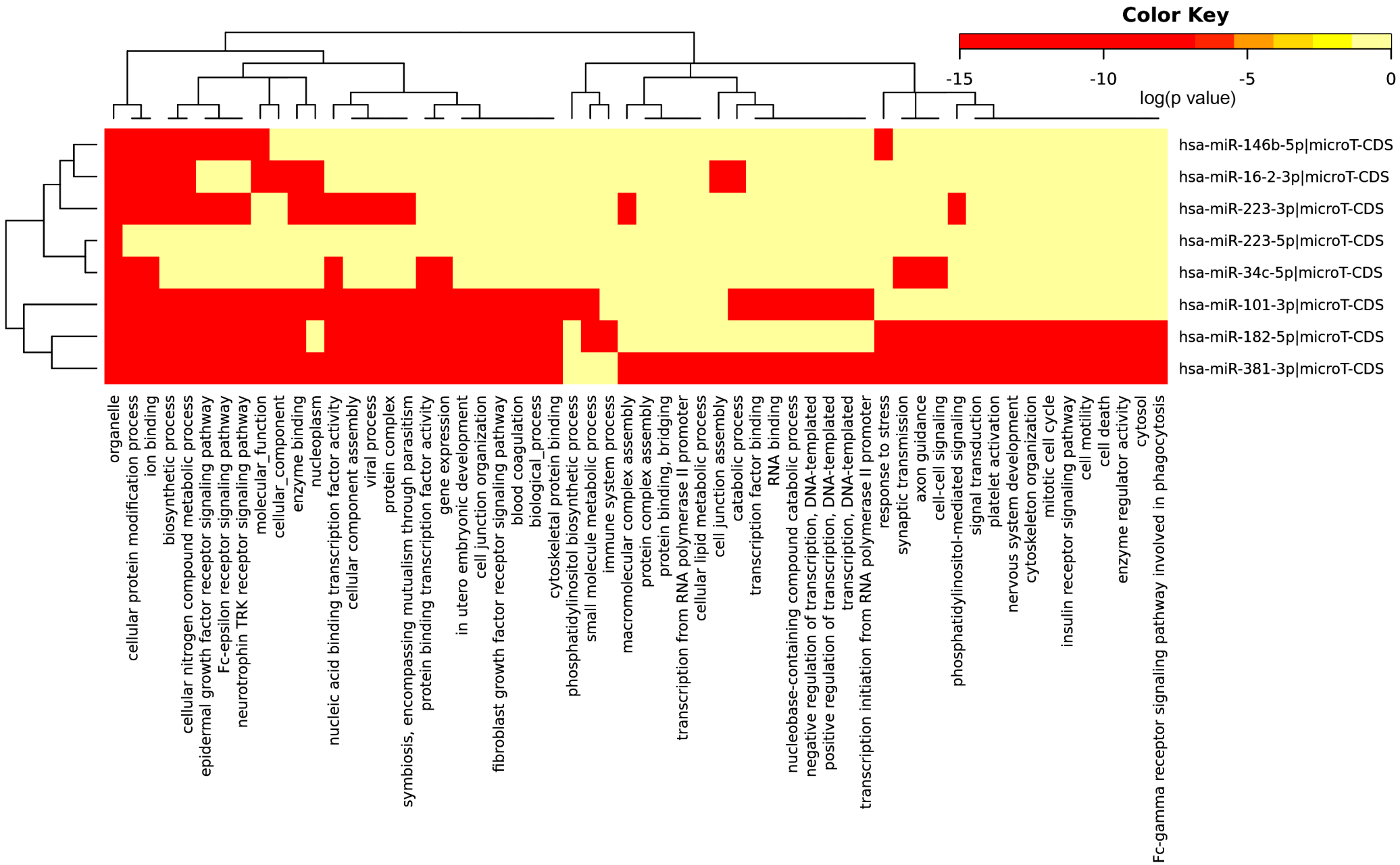


Supplementary Figure S5, Interactive heat maps showing the KEGG enriched pathways targeted by the miRNAs


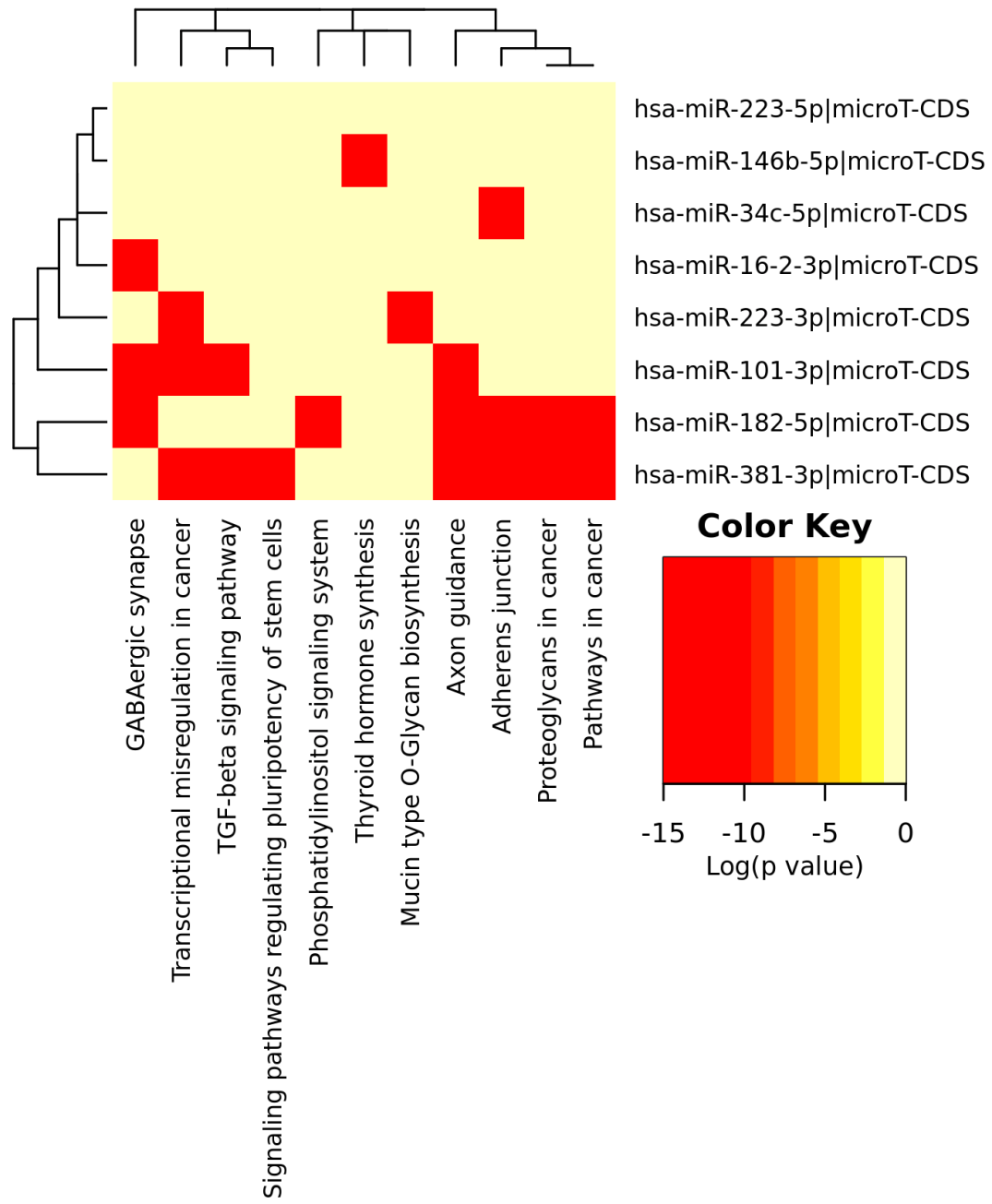

Supplement: Supplementary file 1 [file Data_Sheet_1.docx]
